# Sweet vs. Salty Former Food Products in Post-Weaning Piglets: Effects on Growth, Apparent Total Tract Digestibility and Blood Metabolites

**DOI:** 10.3390/ani11113315

**Published:** 2021-11-19

**Authors:** Alice Luciano, Marco Tretola, Sharon Mazzoleni, Nicoletta Rovere, Francesca Fumagalli, Luca Ferrari, Marcello Comi, Matteo Ottoboni, Luciano Pinotti

**Affiliations:** 1 Department of Health, Animal Science and Food Safety, VESPA, University of Milan, Via Trentacoste 2, 20134 Milan, Italy; marco.tretola@agroscope.admin.ch (M.T.); sharon.mazzoleni@unimi.it (S.M.); nicoletta.rovere@unimi.it (N.R.); francesca.fumagalli1@unimi.it (F.F.); luca.ferrari2@unimi.it (L.F.); matteo.ottoboni@unimi.it (M.O.); luciano.pinotti@unimi.it (L.P.); 2 Agroscope, Institute for Livestock Sciences, 1725 Posieux, Switzerland; 3 Department of Human Science and Quality of Life Promition, Università Telematica San Raffaele, Via di Val Cannuta 247, 00166 Rome, Italy; marcello.comi@uniroma5.it

**Keywords:** former food, pig, circular economy, alternative feed ingredients, blood metabolites, digestibility, growth performance

## Abstract

**Simple Summary:**

Nowadays, researchers need to find a solution to the growing demand for sustainable animal productions. Livestock animal’s nutrition is the component with major impacts on environment and economy. The biggest challenge is to find alternative feed ingredients to minimize and valorize the food leftovers. Food industry leftovers, also called former food products, could be a valid alternative to grains in young pigs’ nutrition. From a nutritional point of view, these ingredients are very similar to standard cereals, like corn. The results from this study suggest that a partial substitution of standard ingredients with two different sources of former food products in the diets of post-weaned pigs is possible, without any negative effects on growth performance and health of animals.

**Abstract:**

Former food products (FFPs) have a great potential to replace conventional feed ingredients. This study aimed to investigate the possibility to partially replace standard ingredients with two different types of FFPs: bakery (FFPs-B) or confectionary (FFPs-C) FFPs and their effects on growth performances, feed digestibility and metabolic status in post-weaning piglets. Thirty-six post-weaning piglets were randomly assigned to three experimental diets (*n* = 12 per diet) for 42 days: a standard diet (CTR), a diet where 30% of standard ingredients were replaced by confectionary FFPs (FFPs-C) and a diet where 30% of standard ingredients were replaced by bakery FFPs (FFPs-B). Individual body weight and fecal dry matter were measured weekly. Feed intake (FI) was determined daily. Average daily gain (ADG), average daily feed intake (ADFI) and feed conversion ratio (FCR) were calculated. Fecal samples were collected daily for three days/week to determine apparent total tract digestibility of dry matter (ATTD). At day 0, 21 and 42, blood samples were collected from all the piglets. No significant differences (*p* > 0.05) between groups were found in growth performances and metabolic profile. However, ATTD in FFPs-B group was lower (*p* < 0.05) compared to the CTR group at the end of the experiment. This study confirmed the possibility to formulate homogeneous diets integrated with 30% of both categories of FFPs. Further investigations are needed to clarify the effects of bakery former food products on the digestibility of the diet.

## 1. Introduction

The loss of natural resources within the food cycle is a global problem. An estimated 1.3 billion tons of food are wasted or lost every year [1]. This amount represents one-third of all that is produced for human consumption [1]. The 2030 Agenda for Sustainable Development reflects the increased global awareness of the problem. One target of the FAO Sustainable Development Goals calls for halving per capita global food waste at retail and consumer levels by 2030, as well as reducing food losses along the production and supply chains [2]. Fighting food waste and increasing the sustainability starting from food production could be a valid contribution, but it is certainly one of the biggest challenges we are called to participate in [3]. In this scenario, animal nutrition researchers are focusing their attention on the use of food losses in animal diets as a valid alternative to standard ingredients like corn or wheat [4]. Indeed, food losses from these food industries are converted into ingredients for the feed industry and are re-entered in the food chain with a circular economy vision [4]. Food losses, also called former food products (FFPs), ex-food, food leftover or bakery meal, have been demonstrated to represent valid and authorized [4,5,6] ingredients from a nutritional point of view for both monogastrics and ruminants [7]. The practice to replace cereals or other standard ingredients with FFPs in animal diets is increasing thanks also to the rising knowledge about their properties. In this sense, the inclusion of FFPs in feed and in the livestock supply chain is a good compromise for improved livestock sustainability [7]. For a better investigation of the FFPs properties, these products have been classified into two main categories: bread, pasta and salty snacks from bakery production (FFPs-B) and chocolates, biscuits and sweet snacks from confectionary production (FFPs-C) [7]. Recent studies analyzed FFPs for their nutritional, functional, chemical composition and digestibility features [4,5,6,7,8,9,10]. They have been defined as a “fortified version of common cereals’’, because of their higher metabolizable energy, fat and starch content on dry matter basis, compared with traditional feedstuffs. In vitro studies on carbohydrate digestibility showed that, compared to common cereals, FFPs are characterized by a higher digestibility potential due to the presence of readily available simple sugars and processed starch [4,5,6,7,8,9,10,11,12]. The high digestibility of FFPs is probably related to the industrial processes they undergo such as heat and mechanical treatments. The processing led to a starch gelatinization which facilitates the enzymatic hydrolysis of starch increasing the product digestibility. On one hand, the high digestibility of FFPs can be considered an advantage, especially for young animals characterized by a lower ability to digest nutrients compared to older animals [9]. On the other hand, gut health could be affected by the lower amount of undigested particles that can reach the large intestine as substrate for bacterial growth. The high predicted glycemic index (pGI) observed when FFPs were included in a complete diet for monogastric animals [8] could result in a potential more rapid return to a state of hunger of the animal, improving the feed intake. Concerns could be related to the nutritional diarrheas due to the high concentration of simple sugars, which may disturb the osmotic balance across the enteric epithelium, causing excessive water secretion and loose stools [10]. Therefore, more information about digestibility, pGI and other properties of diets including FFPs is needed to improve the formulation of more accurate and balanced diets for young pigs that must adapt their digestive enzymes to new alternative dietary components [11]. Filling the lack of knowledge about the effects of FFPs on animal performances and health is essential to increase their use in animal nutrition for a new and sustainable livestock nutrition. A recent study on post-weaning piglets demonstrated that FFPs can partially substitute conventional ingredients without detrimental effects on apparent total tract digestibility (ATTD), growth performance or hematological parameters in the short period (16 days) [9]. Another study tested co-products from rice milling industry in diets for pig weanling, resulting in an improved growth performance of the animals fed experimental diets [11]. In a similar trial, co-products including rice hulls, rice bran and broken rice were included in diets for nursery pigs with no damaging effects on growth performance [12]. To our knowledge, no studies investigated the effects of high inclusion (more than 30%) of sweet and salty FFPs in piglets’ diets during the entire post-weaning period. Based on all the above-mentioned information, the objective of this study was to partially replace common ingredients (30% on dry matter basis) with FFPs in pig diets and to evaluate the growth performance, ATTD and blood metabolites in piglets at the early (21 days) and late (42 days) post-weaning period using the same diet.

## 2. Materials and Methods

The protocol for this experiment was reviewed and approved by the Animal Care and Use Committee for Livestock of the University of Milan, OPBA (Organismo Preposto al Benessere Animale) and received the authorization from the Italian Ministry of Health (N° 405/2019-PR). Moreover, the principles of the 3R (Replacement, Reduction and Refinement) were applied to the trial authorized by the Italian Ministry of Health. The trial was conducted at the Experimental Animal Research and Application Center in Lodi (LO), at the University of Milan.

### 2.1. Animals and Experimental Design

Thirty-six post-weaning female piglets (Large White × Landrace pigs −28 days of life, 6.5 ± 1.1 kg) were selected from a breeding farm in the north of Italy. Animals were housed in individual pens in the same room and same environmental conditions, with controlled temperature and air speed. Each pig was able to interact with other subjects according to the regulations on animal welfare. According to a European Directive [13] (EC Directive 2008/120/EC), environmental enrichment was provided in the form of small plastic balls for kids, completely safe and resistant for piglets.

The piglets were randomly grouped to obtain similar conditions of initial body weight in piglets fed with the standard post-weaning diet (CTR), salty (bakery) former food products (FFPs-B) and sweet (confectionary) former food products (FFPs-C) as described below. All pigs always had *ad libitum* access to water. After 7 days of adaptation, pigs received the three experimental diets *ad libitum* for 42 days. At the end of the experiment, six pigs per group were slaughtered to collect samples used in parallel investigations.

### 2.2. Experimental Diets

The chemical composition of the three diets was analyzed according to the Association of Official Analytical Chemists (AOAC) and the European Commission N° 152/2009 [14]. Piglets belonging to the CTR group were fed a standard diet for post-weaning piglets. Piglets belonging to the FFPs-B and FFPs-C groups were fed diets in which the 30% of conventional ingredients were replaced by bakery and confectionary FFPs, respectively. Table 1 resumes the chemical composition of the pure FFPs-B and FFPs-C products used to formulate the complete experimental diets. The nutrient composition of the three experimental diets met NRC [15] requirements and were iso-energetic (14.0 MJ/kg DM) and iso-nitrogenous (19.0% DM).

The FFPs and the complete diets were provided mixed in mash form and prepared by two FFP processing companies based in the north of Italy. Table 2 reports the ingredients composition of the three complete diets used in this trial.

The analyzed chemical composition of the three experimental diets is reported in Table 3.

### 2.3. Growth Performance

Individual pig body weight (BW) and fecal dry matter were measured weekly, while feed intake (dFI) was recorded daily. In addition, average daily gain (ADG), average daily feed intake (ADFI) and feed conversion ratio (FCR) were calculated. These measurements were carried out to evaluate the growth performance of the animals in relation to the different experimental diets.

### 2.4. Apparent Total Tract Digestibility (ATTD) of Dry Matter

For the determination of the apparent total tract digestibility of dry matter (ATTD of DM), fresh fecal samples were collected daily before the morning feeding for three consecutive days for each week.

The ATTD of DM was determined using the acid-insoluble ashes (AIA) method [16]. Acid-insoluble ash (AIA) is considered a neutral marker for determining digestibility of feed in pigs and seems more suitable than metal elements like iron or chromium [17]. The amount of natural occurring AIA in the feed was verified in all experimental diets in order to avoid the inclusion metal elements as indigestible markers. The amount of natural occurring AIA was above 4 g·kg^−1^ feed in all experimental diets, which is considered more than adequate for the estimation of ATTD in pig [16,17]; accordingly, non-inclusion of supplemental AIA sources was performed. After being collected, the feces were stored in plastic containers and frozen at −20 °C until the time of analysis. The samples collected in the three consecutive days within the week were analyzed as a single pool. The feces were weighed in a 50 mL crucible and then dried in a ventilated oven at 80 °C for 48 h, cooled in a desiccator to room temperature, reweighed and then incinerated in a muffle at 450 °C. The ash was placed in a beaker to which 100 mL of HCl 4N was added. The compound was boiled for 5 min on a stove-top. Then, the hydrolyzed compound was filtered, and the filter was rinsed to remove the acid with hot distilled water (85–100 °C). The filter and the ash content were placed in the original crucibles and then they were incinerated in a muffle at 450 °C. Finally, the crucibles with the contents were cooled in a desiccator at room temperature, weighted with the ash content (Wf) and weighted again after being emptied (We). The percentage of insoluble acid ash was calculated using the equation

AIA = (Wf − We)/Ws × 100

where Wf = weight of the crucible with the ashes, We = weight of the empty crucibles and Ws = weight of the dry matter sample.

The ATTD of DM was calculated according to the indirect digestibility method [16], as follows:

ATTD (g/100 g DM) = (1 − A/B) × 100

where A and B were the AIA concentrations in the feed and feces, respectively.

### 2.5. Blood Samples and Metabolic Profile

During the trial, all piglets were fasted over the night before blood collection at day 0, 21 and 42. In total, 54 blood samples were collected from jugular vein. Blood was collected using Vacutainer EDTA tubes and immediately centrifuged at 14,000 rpm for ten minutes at room temperature to obtain plasma. The plasma was then collected and frozen at −80 °C in the presence of protease inhibitors, for further determine the following metabolites: Total proteins, Albumin, Globulin, Albumin/globulins (A/G), Urea, Alanine aminotransferase (ALT-GPT), Aspartate aminotransferase (AST-GOT), Alkaline phosphatase (ALP), Total bilirubin, Glucose, triglycerides, amylase, total cholesterol, calcium, phosphorus and magnesium. Next, blood samples were sent to external laboratory to be analyzed. These parameters were measured through a standard enzymatic colorimetric analysis using a multiparameter autoanalyzer for clinical chemistry (Instrumentation Laboratory Company, Lexington, MA, USA).

### 2.6. Statistical Analysis

Data were analyzed using IBM SPSS Statistics version 27 (SPSS, Chicago, IL, USA). Single pig was considered the experimental unit. Data were tested for normality with the Shapiro–Wilk test before statistical analysis and boxplot analysis was conducted in order to detect and delete outliers. Growth performance data (BW, ADFI, ADG and FCR), ATTD of dry matter and plasma biochemical values were analyzed using one-way analysis of variance (ANOVA) in order to compare means. The REPEATED statement was used for variables measured over time (BW, ADFI, ADG, FCR, ATTD and plasma metabolites). Differences with *p* values < 0.05 were considered to be significant. The analysis was performed using the following model:

*yij* = *μj* + *εij*

where *yij* is the observation (values), *μj* is the mean of the observations for the *j*-th group (sample) and *εij* represents the within-sample random variability. Differences with *p* values < 0.05 were considered significant. Data are presented as means ± Standard error mean.

## 3. Results

### 3.1. Growth Performance and Apparent Total Tract Digestibility of Dry Matter

All animals remained in good health throughout the experiment and there were no morbidity or mortality issues. No effect between experimental group and period was observed for all the growth performance parameters. Accordingly, data were presented as week 1, week 5 and the overall mean of experimental period. The results of the present study regarding pigs’ growth performance showed that there were no significant differences in BW between groups (*p* > 0.05) (Table 4). Body weight measured at the piglets’ arrival and at the end of the trial did not differ between diets (Table 4). The body weight of the pigs increased regardless of the diet treatments, without showing any statistically significant differences between groups at the same time point (Table 4). The ADFI was not affected (*p* > 0.05) by any dietary treatments (Table 4). In addition, the experimental diets did not influence (*p* > 0.05) the ADG and feed conversion ratio (FCR) (Table 4) either. The results revealed that initial ATTD of DM values did not differ (*p* > 0.05) between the CTR and the two experimental diets. However, the final values of the ATTD of DM showed that final ATTD of DM of CTR diet is similar to the one of FPPs-B diet, but the ATTD of DM of FFPs-C diet is lower compared to the final ATTD of DM of the CTR and FPPs-B diets (*p* < 0.05).

### 3.2. Blood Samples and Metabolic Profile

All the metabolites analyzed in the plasma of pigs are reported in Table 5. No significant effects of the diets (*p* > 0.05) were found in the analyzed hematological parameters between groups over the entire experiment (data not shown). Accordingly, the overall mean values were reported (Table 5).

## 4. Discussion

### 4.1. FFPs Use in Post-Weaning Pig Diets and Composition

The use of FFPs is still limited due to the lack of knowledge regarding the effects of those products on growth performance and animal wellbeing [4]. One concern could be the difficulty to formulate a standard feed due to the variability of ingredients used for the FFPs production [7]. However, in USA, a study conducted on 46 sources of bakery meal collected from the 6-state area reported that the differences among geographical regions in the chemical composition of bakery meals were small and only the concentration of ash and some other nutrients differ between the different area [18]. Former food producers can predict the variations between the different products’ storage and guarantee a final product with a standardized composition, due to different process of homogenization [7]. This study showed that the 30% inclusion of two different types of FFPs in the diets of post-weaning piglets is possible. Based on the nutritional facts reported for humans, FFPs are rich in carbohydrates and fat, depending on their origin. For this reason, FFPs are commonly used in young animals, especially post-weaning pigs and calves [7]. Similarities between the chemical composition of common cereals and different FFPs have been already demonstrated [4,6,7,8], while a higher glycemic index potential in FFPs compared to corn and heat processed wheat has been observed in vitro [8]. As reported in Kaltenegger et al. [19], FFPs have a high nutritional value for animal feed because of their high energy content. In this study, the ME of two types of FFPs diets was ~14.0 MJ/kg. These values are in line with the literature [20], where the digestible energy in bakery meal diet was 14.0 MJ/kg was similar compared to the standard diet with corn that was 14.2 MJ/kg. The high energy content of the FFPs products make them a valuable opportunity to replace other energy-rich ingredients traditionally used for feed formulation, with positive effects on the circularity of the food production.

Regarding the chemical composition and nutritional values, the FFPs-C and FFPs-B diets are comparable with other studies [7,8,9,10,11,12,13,14,15,16,17,18,19,20,21]. One of the main concerns about this material is its homogeneity and stability in composition over the time. It is correct that a wide range of different ingredients for the production of the final product could be used. An example is the effect of the seasonality, where at some times of the year (e.g., Easter, Christmas, etc.) a large amount of sugary products could be available, instead of other products. However, FFPs processors are able to assure a final product with no significant variations in the chemical composition. This result is obtained by stocking different ingredients singularly, analyzing their chemical composition and, based on the results, and mixing them in a way to obtain a final product with similar characteristics all over the year [4]. In the current study, the two FFP-based diets were characterized by comparable amount of starch and NDF contents in respect of the CTR diet. Has been showed that the starch and fiber content of different categories of FFPs can vary based on the main sources of ingredients used for their production [4]. As expected, a difference between the three diets is the content of simple sugar, higher in FFPs-C compared to FFPs-B and CTR. In the previous study of Tretola et al. [9], only one source of FFPs containing both bakery and confectionary products was tested. Because of the intestinal health implication related to the sugar content in the pig’s diet, a categorization of the FFPs based on their ingredients composition (e.g., sugary vs. salty) could result in an easier diet formulation.

### 4.2. Growth Performance

During weaning, piglets are exposed to a wide range of stressors such as the change of environment, transition from a liquid to a solid diet and immature digestive system that may could lead to a depression in growth rate [9,21,22]. The inclusion of processed food such as FFPs could represent an additional stressful factor for the weaned piglets on one hand or a source of more readily available nutrients for the still immature intestine on the other hand. In this study, the use of FFPs in post-weaning diet did not affect the growth performance of the piglets. In particular, body weight, ADFI, ADG and FCR were similar between the three groups. Those results confirm what has been already observed in a similar study, where no detrimental effect on growth performance have been observed in pigs fed FFPs-based diet [9]. Similarly, Rojas et al. [20] did not find any differences in feed intake between diets composed by bakery meal and standard diet with corn (473 g DM/d and 481 g DM/d, respectively). Another study investigated the effect of candy co-products as an alternative source of carbohydrates and lactose in newly weaned pigs [21]. The results showed that candy co-products could replace up to the 45% of dietary lactose without compromising growth performance, feed intake and dietary efficiency. In particular, ADFI, ADG and gain to feed ration did not have any statistical change if whey permeate was substituted with candy coproduct [21]. More recently, Luciano et al. [22] have investigate the effect of bakery meal as corn substate (substitution rate from 25% up to 100%). Results indicated that for the overall 5-wk nursery period, increasing concentrations of bakery meal above 30% (i.e., corn substitution rate of 50%) tended to reduce average daily gain and reduced gain to feed ratio of pigs, whereas blood indicators of energy and protein utilization were not affected. Specifically, there was no effect of increasing concentrations of bakery meal on growth permeance of pigs from day 1–14. However, ADG of pigs from day 15–35 and for the overall experimental period tended to decrease as the concentration of bakery meal increased in the diets above 30% on dry matter basis. The G:F from day 15–35 and for the overall experimental period linearly decreased as bakery meal inclusion increased in the diets. However, no differences among dietary treatments were observed from day 15–35 or for the overall experimental period for feed intake and the final body weight on day 35. These results [22] are in line with the present study, confirming that both FFP-C and FFP-B do not represent any issue to formulate balanced and homogeneous diets for piglets during weaning up to 30% of inclusion. Considering that the weaning is the most critical stage of the pig’s life, the lack of detrimental effects in piglets should also support the use of those ingredients in finishing pig’s diets, when the feed intake is higher and the potential mitigation of the environmental impact increased.

### 4.3. Apparent Total Tract Digestibility (ATTD) of Dry Matter

Confectionary and bakery products used for the production of FFP-C and FFP-B, respectively, are subject to numerous technological processes that can improve their digestibility. While the ATTD of DM of piglets fed FFPs-B was similar to that of the CTR group, FFPs-C decreased the ATTD of DM, in contrast with our expectations. In previous experience, the partial replacement of cereals with FFPs resulted in an increased ATTD, with ATTD values of 83% and 78% for FFPs and CTR diets, respectively. Food processing, in fact, often results in small food particles which have a greater surface in contact with digestive enzymes compared to coarser ones, leading to a greater digestion rate [23]. Heat is another factor that influences feed digestibility, where high-temperature treatments can improve digestibility values by the protein denaturation of some anti-nutritional elements such as the anti-tryptic factor of raw soybeans [24]. Extrusion significantly increases the digestibility of starch for both humans and animals [25]. It is widely known that piglets benefit from the consumption of easily digestible carbohydrates until their digestive system is fully able to use the starch. Thus, it is not clear why the FFPs-C resulted in a decreased ATTD of DM. One hypothesis could be the balance between simple sugars and starch content of the diet. The ratio between simple sugars/starch was 0.17 for FFPs-C diet, while it was ~0.11 for the other two diets. In this context, the role of fiber needs to be addressed too [26]. The rates of sugar absorption depend on the form in which they are consumed and also on the effects of individual food matrices on gastric emptying [27]. A second hypothesis could be associated to the processing of bakery products itself. The fast intestinal transit of the small processed food particles through the intestinal tract can negatively affect their digestibility due to a reduced contact time with digestive enzymes [23]. Therefore, the right balance between a particle’s size and its transit speed through the gastrointestinal tract is essential to obtain the highest digestibility value. We could also speculate that sugary ingredients used in FFP-C, such as chocolate products, were richer in tannins compared to the ingredients used in CTR and FFP-B. Even if the content of tannins has not been quantified in this study, it is known that the content of tannins in cocoa products is higher compared to cereals [28]. Tannins are polyphenolic biomolecules that can interact with and precipitate macromolecules, such as proteins, gelatins, polysaccharides and alkaloids [29]. Therefore, the interaction of tannins from cocoa products with nutrients and digestive enzymes could have led to a reduced digestion rate in FFP-C group.

### 4.4. Blood Metabolic Profile

Diet has a measurable and significant effect on blood components [30]. The analysis of hematological parameters represents a readily available assessment of the health status of animals during a feeding test and at the same time can be used as an appropriate measure of nutritional status [31]. In the current study, no differences in blood metabolites between the two FFPs diets compared with CTR diet were found. This result is in line with our previous experience, where FFPs did not significantly affected the selected blood parameters, but increased glucose and decreased urea concentration compared to the CTR group [9]. The high potential glycemic index of FFPs due to the high content of simple sugar and processing-related characteristics of the starch has been already described [9]. However, in the present study no differences in the glycaemia were observed between the three experimental diets. These findings could be related to several aspects and characteristics of FFPs. First, only the FFPs-B diet had a higher sugar content compared to the CTR diet. However, its starch content was lower. These differences in the chemical composition could have led to a balanced contribution to the glycemic index, resulting in similar values. Another explanation could be associated to the easily available sugars in the FFPs diets. As described by Ottoboni et al. [8], the highest amount of sugar content by FFPs is released from the matrix within the first 15 min after digestion, while in the following time points the glucose release is similar to the standard cereals. In this study, blood samples for the serum metabolites investigation were collected after 8 h of fasting, when tissues probably already metabolized the blood glucose, restoring the glycemia to baseline values independently by the diet. Post-prandial blood sampling in further research could confirm the potential of FFPs to increase pig glycemia and therefore potentially increase the hanger status of the animals, even if the similar feed intake observed between groups suggest a lack of significant effects in this respect. The results obtained in this study suggest that the introduction of 30% FFPs into the feed mixture for weaned piglets does not affect the metabolic profile of the animal, under the condition that the nutrients and metabolizable energy in the mixture are properly balanced and cover the animal requirements.

## 5. Conclusions

The results obtained in this study suggest that 30% of FFPs can be included in post-weaning pig diets as alternative ingredients to improve sustainability in the livestock sector. Moreover, the two types of bakery or confectionary FFPs are completely comparable to the blend of FFPs used in previous study and conventional diet. However, further investigations are necessary to clarify the reason why FFPs-C decreased the ATTD of DM and to evaluate the effects of FFPs obtained by confectionary or bakery companies on other parameters like carcass composition and gut health.

## Figures and Tables

**Table 1:** Analyzed composition (g/100 g or MJ/kg on DM) of the two pure former food products used for the FFPs-C and FFPs-B experimental complete diets in post-weaned piglets.

Item	Pure FFPs-C ^1^	Pure FFPs-B ^2^
DM	91.0	87.7
DE (MJ/kg)	19.6	19.4
CP	10.0	11.0
Ash	2.10	2.10
Crude Fats (after hydrolysis)	9.59	7.50
CF	1.60	2.20
Starch	42.5	50.5
NFE	67.80	64.9
TS (expressed in sucrose)	21.0	10.5
Fe (mg/kg)	41.7	95.0
Sodium chloride	0.2	0.15
Amino acids		
Arg	0.48	0.20
His	0.19	0.17
Ile	0.33	0.27
Leu	0.59	0.68
Lys	0.26	0.18
Met	0.05	0.13
Phe	0.40	0.50
Thr	0.25	0.31
Val	0.40	0.27
Ala	0.29	0.66
Asp	0.48	0.40
Cys	0.10	0.10
Glu	2.44	2.87
Gly	0.32	0.48
Pro	0.80	1.34
Ser	0.40	0.54
Tyr	0.22	0.19
Total	8	9.29

Abbreviations: DM: dry matter; DE: digestible energy; CP: crude protein; CF: crude fiber; NFE: nitrogen-free extracts; TS: total sugars. ^1^ Pure FFPs-C: Pure confectionary former foodstuff products. ^2^ Pure FFPs-B: Pure bakery former foodstuff products.

**Table 2:** Ingredient composition (g/100 g of diet) of the three diets for post-weaning piglets.

Ingredients	CRT ^1^	FFPs-C ^2^	FFPs-B ^3^
Wheat	25	25	17
Pure FFPs-C	-	30	-
Pure FFPs-B	-	-	30
Wheat flaked and hulled	10	-	-
Barley flaked and hulled	10	-	-
Barley	14.1	6.1	10
Sweet whey	8	8	8
Whole soybeans flaked and ground	6.2	1	4
Wheat Bran	5	14	11
Fermented soy protein concentrate	5	3	5
Rice flakes	5	-	5
Vitamin pre-mix including flavor	2.7	2.7	2.2
Fish Meal	2	2	2
Soybean Meal 47%	1.4	4.9	-
Soybean Oil	1.3	-	1.7
Sucrose	1	-	1
L-Lysine	0.7	0.8	0.9
Monocalcium phosphate	0.5	0.4	0.8
Calcium carbonate	0.5	0.5	0.5
Sodium chloride ^4^	0.5	0.5	0.5
L-threonine	0.3	0.4	0.4
DL-methionine	0.3	0.4	0.5
B vitamins	0.2	0.2	0.2
L-valine	0.2	0.3	0.3
L-tryptophan	0.1	0.1	0.1

^1^ CRT: control diet. ^2^ FFPs-C: confectionary former food products diet. ^3^ FFPs-B: bakery former food products diet. ^4^ Sodium chloride added in order to reach 0.5 g/100 g in each diet.

**Table 3:** Analyzed composition (g/100 g on dry matter basis) of the three diets for post-weaning piglets.

Item	CTR ^1^	FFPs-C ^2^	FFPs-B ^3^
DM	90.1	90.2	88.9
CP	19.1	19.1	19.0
NSC	57.6	59.1	58.4
Ash	6.11	6.10	6.19
Crude fat	3.90	3.99	3.71
Starch	39.9	38.0	39.7
NDF	11.2	10.7	9.7
ADF	3.71	3.42	3.23
Simple Sugar	4.69	6.60	4.70
Ca	0.7	0.7	0.7
P	0.6	0.6	0.6
Lys	1.5	1.5	1.5
Met	0.6	0.6	0.8
ME (MJ/kg)	14.0	14.0	14.0

Abbreviations: DM: dry matter; CP: crude protein; NSC: Non-structural carbohydrates; NDF: neutron detergent fiber; ADF: acid detergent fiber. ^1^ CTR: control diet. ^2^ FFPs-C: confectionary former food products diet. ^3^ FFPs-B: bakery former food products diet.

**Table 4:** Effects of partial replacement of conventional ingredients by FFPs-B and FFPs-C on growth performance of post-weaning piglets.

Item	CTR ^1^	FFPs-C ^2^	FFPs-B ^3^	SEM	*p*-Value
Week 1 (0–7 d)					
Initial body weight (kg)	6.85	6.64	6.62	0.25	0.65
ADFI (kg)	0.23	0.21	0.18	0.02	0.55
ADG (kg)	0.18	0.18	0.13	0.02	0.62
FCR (kg/kg)	1.63	1.45	1.63	0.23	0.62
ATTD of DM (g/100 g DM)	82.3	80.7	82.1	2.27	0.76
Week 5 (35–42 d)					
Final body weight (kg)	26.2	24.8	24.5	1.17	0.54
ADFI (kg)	1.08	1.01	1.03	0.06	0.70
ADG (kg)	0.75	0.75	0.74	0.04	0.99
FCR (kg/kg)	1.48	1.44	1.39	0.09	0.79
ATTD of DM (g/100 g DM)	89.4 ^a^	86.1 ^b^	90.3 ^a^	1.11	0.002
Overall mean (0–42 d)					
Body weight (kg)	14.9	13.8	14.1	0.43	0.56
ADFI (kg)	0.69	0.64	0.63	0.02	0.59
ADG (kg)	0.47	0.44	0.45	0.01	0.62
FCR (kg/kg)	1.49	1.50	1.48	0.02	0.96
ATTD of DM (g/100 g DM)	86.3	86.7	84.4	0.46	0.10

Data presented are the means of *n* = 12 replicates/group with their standard errors at week 1, week 5 and for the overall experimental period. Abbreviations: BW: body weight; ADFI: average daily feed intake; ADG: average daily gain; FCR: feed conversion ratio; ATTD: apparent total tract digestibility. ^1^ CTR control diet. ^2^ FFPs-C: diet with confectionary former food products. ^3^ FFPs-B: diet with bakery former food products. ^a,b^ Values within a row with different superscripts differ significantly at *p* < 0.05.

**Table 5:** Effects on overall mean values of blood metabolites of partial replacement of conventional ingredients by FFPs-B and FFPs-C of post-weaning piglets.

Item	CTR ^1^	FFPs-C ^2^	FFPs-B ^3^	SEM	*p*-Value
Total proteins (g/L)	46.8	47.2	46.9	1.29	0.94
Albumin (g/L)	27.8	26.3	26.6	0.99	0.31
Globulins (g/L)	19.2	20.9	20.4	1.22	0.36
Urea (mmol/L)	1.63	1.91	1.76	0.24	0.50
ALT-GPT(IU/L)	46.4	47.3	46.9	3.91	0.97
AST-GOT (IU/L)	56.7	50.0	59.4	5.38	0.21
ALP (mmol/L)	229	204	226	22.2	0.49
Bilirubin (mmol/L)	1.64	1.69	1.64	0.12	0.88
Glucose (mmol/L)	6.34	6.17	5.79	0.27	0.13
Cholesterol (mmol/L)	2.06	2.07	2.04	0.13	0.96
Calcium (mmol/L)	2.84	2.82	2.75	0.07	0.41
Phosphorus (mmol/L)	3.13	3.06	3.06	0.11	0.76
Magnesium (mmol/L)	0.97	0.94	0.96	0.02	0.47
Amylase (mmol/L)	2092	1663	1598	89.7	0.06
Triglycerides (mmol/L)	0.43	0.50	0.47	0.01	0.16

Data presented are means of *n* = 12 replicates/group with their standard errors. For each diet, 12 observations per sampling time were considered. Abbreviations: ALT: alanine aminotransferase; GPT: glutamate pyruvate transaminase; AST: Aspartate amino transferase; GOT: glutamate-ossalacetate transaminase; ALP: Alkaline phosphatase. ^1^ CTR: control diet. ^2^ FFPs-C: diet with confectionary former food products. ^3^ FFPs-B: diet with bakery former food products.

## Data Availability

The data that support the findings of this study are available from the corresponding author upon reasonable request.
